# Mycobacteriosis and Infections with Non-tuberculous Mycobacteria in Aquatic Organisms: A Review

**DOI:** 10.3390/microorganisms8091368

**Published:** 2020-09-07

**Authors:** Mohammad Reza Delghandi, Mansour El-Matbouli, Simon Menanteau-Ledouble

**Affiliations:** Clinical Division of Fish Medicine, University of Veterinary Medicine, Veterinärplatz 1, 1210 Vienna, Austria; mohammad.delghandi@vetmeduni.ac.at (M.R.D.); mansour.el-matbouli@vetmeduni.ac.at (M.E.-M.)

**Keywords:** *Mycobacterium marinum*, *Mycobacterium fortuitum*, *Mycobacterium chelonae*, Granuloma, chronic infections, diagnostic

## Abstract

The *Mycobacteriaceae* constitute a family of varied Gram-positive organisms that include a large number of pathogenic bacteria. Among these, non-tuberculous mycobacteria are endemic worldwide and have been associated with infections in a large number of organisms, including humans and other mammals and reptiles, as well as fish. In this review, we summarize the most recent findings regarding this group of pathogens in fish. There, four species are most commonly associated with disease outbreaks: *Mycobacterium marinum,* the most common of these fish mycobacterial pathogens, *Mycobacterium fortuitum*, *Mycobacterium gordonae*, and *Mycobacterium chelonae*. These bacteria have a broad host range: they are zoonotic, and infections have been reported in a large number of fish species. The main route of entry of the bacterium into the fish is through the gastrointestinal route, and the disease is associated with ulcerative dermatitis as well as organomegaly and the development of granulomatous lesions in the internal organs. *Mycobacteriaceae* are slow-growing and fastidious and isolation is difficult and time consuming and diagnostic is mostly performed using serological and molecular tools. Control of the disease is also difficult: there is currently no effective vaccine and infections react poorly to antibiotherapy. For this reason, more research is needed on the subject of these vexing pathogens.

## 1. Introduction

Infections caused by members of the *Mycobacterium* genus are common throughout the animal kingdom, including in aquatic animals. However, despite their frequency and prevalence, they have only been the subject of comparatively limited research. The present review, therefore, aims at providing an easily accessible and up to date overview of mycobacteriosis in aquatic organisms (the most recent review, published by Hashish et al., was published in 2018 and focused on *Mycobacterium marinum*) [1]. In particular, we aim at helping with the diagnostic of the disease by making clinical practitioners more aware of its common occurrence, as well as provide a list of up to date methods for its diagnostic. Moreover, we also mention the potential of autogenous vaccines in the prevention of the disease. Finally, we highlight gaps in our understanding of these infections, and suggest avenues for future research.

## 2. Classification and History of the Disease

*Mycobacterium* spp. belong to the family *Mycobacteriaceae* of the order Actinomycetales. These aerobic, non-motile pleomorphic bacilli are Gram-positive despite their mycolic cell wall only fixing the Gram stain poorly, and are usually stained using the Zeihl–Neelsen procedure [2]. The first report of *Mycobacterium* sp. in fish followed its isolation from granulomatous lesions in common carp (*Cyprinus carpio*) in 1897. This bacterium was named *Mycobacterium piscium* and has since been reported from frogs and several other animal species [3,4]. Moreover, multiple other bacterial species have since been described in association with very similar diseases, formerly known as “fish tuberculosis”, although the term is now considered improper, as fish do not develop true tubercles.

The first isolation of *Mycobacterium chelonae* was from two sea turtles (*Chelona corticata*) with pulmonary disease in 1903 [5] while, on the other hand, *Mycobacterium fortuitum* was first isolated in 1953 from neon tetra (*Paracheirodon innesi*) [6]. Lescenko et al. identified *M. avium* subspecies *hominissuis* in ornamental fish, Cockatoo Dwarf Cichlid (*Apistogramma cacatuoides*) with granulomas on the skin [7]. More recently, *M. avium* was isolated from epaulette shark in public aquarium in the Netherland [8]. *M. shottsii* and *M. pseudoshottsii* have been frequently reported from striped bass (*Morone saxatilis*) [9]. In 2017, a new *Mycobacterium* sp. was reported from the thread-sail filefish (*Stephanolepis cirrhifer*) and called *Mycobacterium stephanolepidis* [10]. Moreover, other species have been isolated from other fish species in association with mycobacteriosis (Table 1).

The nomenclature of the various species has been revised over time, and the species *‘Mycobacterium platypoecilus*’, *‘Mycobacterium anabanti*´, alongside *Mycobacterium balnei*, have now been grouped under the name *M. marinum* [11]. Similarly, ´*Mycobacterium ranae*´ has since been reclassified as *M. fortuitum* on the basis of serological (sero-agglutination), physico-chemical profile and lipid pattern [12], while ‘*Mycobacterium borstelence*’ and ‘*Mycobacterium runyonii*´ have been grouped as the species *M. chelonae* [13].

Nowadays, four species of *Mycobacterium* (*M. marinum, M. fortuitum, M. chelonae* and *M. gordonae)* dominate the clinical landscape. In addition, other species can cause disease, notably in ornamental fish, where *M. triviale, M. avium, M. abscessus,* and *M. peregrinum* are regularly reported [14,15,16,17]. This list is still growing as new species are routinely discovered; for example, *Mycobacterium stephanolepidis* that was recently described from diseased farmed thread-sail filefish (*Stephanolepis cirrhifer*) and black scraper (*Thamnaconus modestus*) in Japan [10,18].

These species of non-tuberculous mycobacteria (NTM) can be discriminated based on growth rate and pigmentation: Fast-growing mycobacteria need approximately 7 days to produce colonies on solid agar, but slower growing mycobacteria can need weeks or even months to produce commensurate measurable colonies [19]. This has been explained by the fact that slow-growers have a lengthened helix 18 within the small subunit 16S rRNA molecule, with a size of 3.13–4.29 × 10^9^ daltons and one rRNA (*rrn*) operon per genome. Conversely, fast-growers have a short helix 18 with genome size 4.30–5.20 × 10^9^ daltons and two rRNA (*rrn*) operons per genome (the growth rate is not associated with the number of operons on genome) [20]. *M. marinum* is slow-growing, while *M. fortuitum* and *M. chelonae* are faster growing *Mycobacterium* [21,22].

## 3. Distribution of the Disease

*Mycobacterium* spp. are endemic worldwide, although they appear to be more common in tropical and sub-tropical regions. These organisms have also been reported in colder climates such as Canada [23], Chile [24], Norway [25], and from a variety of aquatic environments in fresh, brackish, and salt-water [26,27], in addition to soil, biofilms and sediments [28,29,30], including the sediment in decorative aquaria and fish breeding facilities [30].

The host range of this disease is correspondingly broad, and includes over 150 species of both marine and fresh water fish, as well as other aquatic organisms such as amphibians and oysters [1]. In addition, NTM have also been isolated from terrestrial animals including mammals and birds as well as reptiles: 28 mycobacterial positive infections have been recognized from 3880 reptile species. For example, *M. marinum* was isolated from a turtle, a bearded dragon and one iguana [31]. *M. marinum*, *M. chelonae*, *M. haemophilum*, and *M. kansasii* are frequently isolated from reptiles [32].

## 4. Course of the Disease and Clinical Signs

Unsurprisingly considering the number of bacterial species involved, these bacteria vary in their pathogenic potential, ranging from true pathogens (*M. marinum, M. ulcerans*), opportunistic pathogens (*M. chelonae–abscessus* complex*, M. fortuitum, M. avium* complex*, M. haemophilum, M. xenopi, M. kansasii* and *M. simiae*) and saprophytes (*M. smegmatis, M. vaccae, M. terrae* complex and *M. gordonae*) [33]. Like most fish pathogens, environmental conditions and stress to the fish often play an important role in the development of the infection, and the most important factors associated with outbreaks include stocking density and low water quality, notably low pH and low dissolved oxygen [28].

Transmission in fish can occur both horizontally [3,34] and vertically, as transovarian transmission has been described in viviparous fish [3,35]. Presently, the three best established hypotheses regarding the horizontal transmission of this disease all involve an oral-enteric route: ingestion of contaminated food, cannibalism of infected fish or infection via sites of injuries and feeding on environmental debris [2,36,37], and the gastrointestinal tract is considered the main site of entrance in zebrafish [38]. Wood and Ordal have reported 100% prevalence of the infection in salmon fry and fingerlings from Pacific Northwest hatcheries following the feeding of contaminated and unpasteurized ground fish meal [39]. Similarly, transmission by feeding infected fish tissue has been demonstrated by Hedrick et al. and Mutoji [40,41]. Furthermore, environmental protozoans graze on bacterial biofilms, and can become infected with *Mycobacterium* and act as vector for the bacterium, protecting and increasing the survivability of these bacteria. Indeed, several outbreaks of mycobacteriosis have been traced back to infected live feed, in particular tubificid worms (*Tubifex tubifex*) and water fleas (*Daphnia* spp.) [42,43,44,45]. Similarly, mosquito larvae infected with *M. marinum* have been associated with infections in medaka fed with these larvae [41].

Infections by NTM develop slowly, over 2 weeks following introduction in the aquarium or injection of the organism, and are associated with a chronic condition; under these circumstances, mycobacteriosis may not always be associated with clinical signs, and mortality may reach 50% with no external signs [3,46]. Moreover, predictably because of the multiple bacterial species involved and the wide range of possible hosts; the clinical signs can vary greatly between infections.

**Figure 1:**
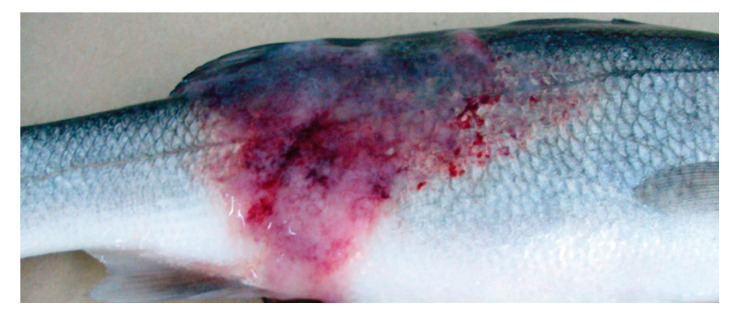
Ulcerative dermal lesions attributed to *Mycobacterium* spp. in striped bass (From Avsever et al. 2016, released under Creative commons [51]).

As is often the case with bacterial infections in fish, the bacteria attach themselves to the basis of the fins, or on skin lesions. Moreover, the earliest external signs will often involve eroded fins and tail rot. In addition, a heavy mucus coating on the body surface, and a change in pigmentation as well as bleaching, have been reported and nonspecific external signs that are often very similar to that of other disease, including swollen abdomen, scale loss, ulcerative dermal necrosis (Figure 1). External red lesions on the lateral line and shallow irregular ulcers have also been reported [1,47,48,49]. Moreover, abnormal behavior, including apathy, loss of appetite and the resulting emaciation can be observed, alongside exophthalmia, blindness, ascites and pale gills, as well as skeletal deformities such as spinal curvature or stunted growth [46,49,50].

**Figure 2:**
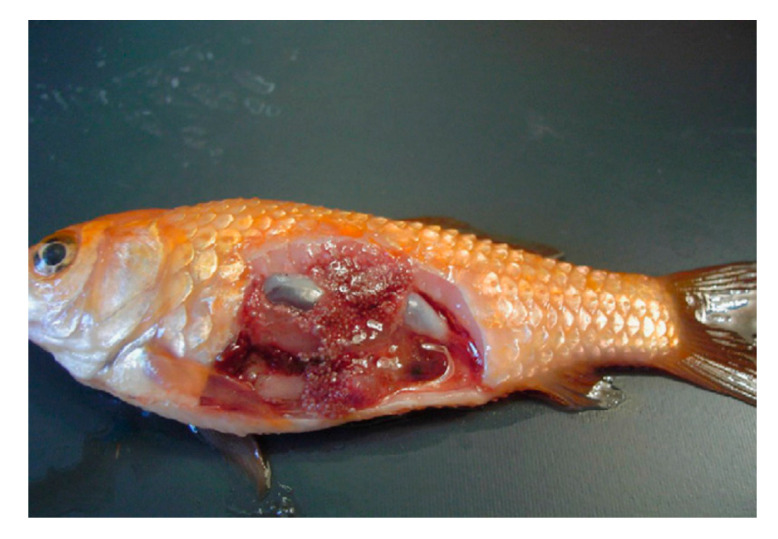
Gray and whitish nodules observed on internal organs of a goldfish (*Carassius auratus*) (From Passantino et al. 2008, reproduced with permission [54]).

Following infection, mycobacterial organisms spread through the whole fish body via the circulatory and lymphatic system [2], and can be found in most tissues and organs, including the eyes, gills, visceral organs, and musculature. Internal signs associated with *Mycobacterium* spp. include organomegaly of the liver, kidney, and spleen. Gray and white nodules in internal organs can be observed occasionally (Figure 2) [2]. Acute disease is characterized by a rapid progress of the infection, uncontrolled growth of the pathogen and death of infected fish within 16 days, whereas chronic infections were defined by the presence of granuloma in different internal organs, and fish can survive approximately 4–8 weeks [1,52,53].

## 5. Zoonotic Consideration

As mentioned above, NTM have been associated with infections in humans. Because aquatic environments are the preferred environments for these bacteria, infection often follows contact with contaminated water or aquatic animals. Consequently, these infections are often associated with the professional activity of the patients [55,56,57]. Similarly, swimming pool infections were very frequent until the 1960s, and these infections were sometimes referred to as “swimming pool granuloma”, however, the occurrence of infections has since been reduced following improvements in swimming pool disinfection [58,59].

Because these bacteria’s thermic preference is lower than the temperature of the human bodies, most infections develop externally and on the body’s extremities. Notably, the disease has been associated with granulomatous lesions, usually on the skin [4,60], which can be painful or not [61], and in some cases can expand into severe necrotic lesions [62]. Commonly, the incubation period lasts around 2–8 weeks, although several cases have been reported with 2–4 months or longer (6–8 months) incubation times [63,64]. More systemic respiratory and extra-respiratory diseases are rare, but can occur, especially in immunocompromised patients [17,65,66,67].

Mycobacteriosis in humans is classified in 4 types (type I–type IV) [1,68]. Type I occurs in patients which are immunocompetent and clinical signs include lesions in superficial tissues with crusted and ulcerates nodules or verrucous plaques. Lesions are small, painless, bluish-red papules 1 to 2 cm in diameter. These signs develop over the course of weeks or months. In type II, lesions with abscesses, inflammatory nodules, and granulomas develop in immunosuppressed patients. Lesions are single or multiple subcutaneous granulomas, with or without ulceration. Type III *M. marinum* infections occur in deep tissues with or without skin lesions. Clinical signs in this category include arthritis, tenosynovitis, osteomyelitis, and bursitis. Type IV of mycobacteriosis is very uncommon, but infection can be found in humans with lung disease [63,69,70].

Intriguingly, studies of the genetic diversity of *M. marinum* between fish and humans isolates have shown difference at the genetic level [71]. Moreover, while *M. marinum* isolated from humans can establish an acute disease in fish, isolates that originated from fish were more often associated with chronic infections [52].

Moreover, the screening of 51 raw milk samples showed that 68.8% of the tested samples were positive for *Mycobacterium* spp., including *M. marinum*, *M. scrofulaceum*, *M. gordonae*, *M. flavescens*, and *M. fortuitum* [72], suggesting that contaminated milk could be an additional route of exposure.

## 6. Diagnostics

### 6.1. Isolation and Cultivation

**Figure 3:**
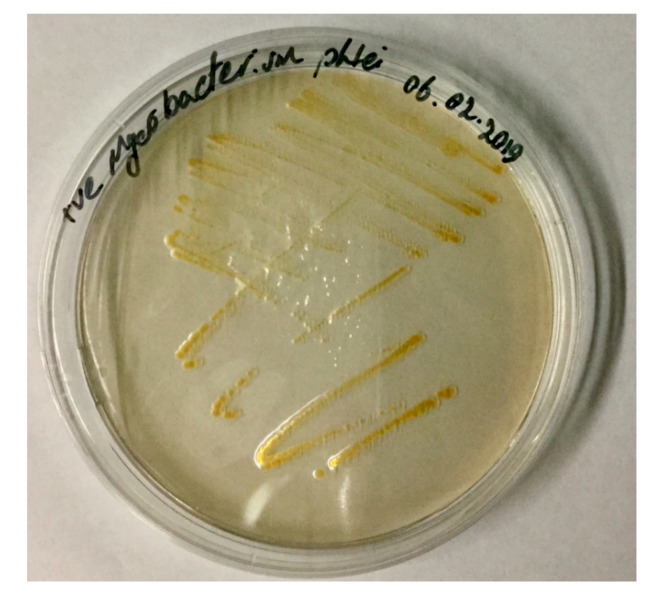
Appearance of *M. phlei* colonies cultivated on Middlebrook 7H10 agar (Picture from Clinical Division of Fish Medicine, University of Veterinary Medicine repository).

Mycobacteria are fastidious and slow growing, and the bacteria are likely to be overgrown by faster growing organisms on non-selective media. These include Middlebrook 7H10 (Figure 3), Löwenstein-Jensen (solid medium), Middelbrook 7H9 (broth medium) growth Petragnani agar and Dorset egg media. Furthermore, *Mycobacterium* withstands treatment with acid and basic chemicals and other combinations as benzalkonium chloride and hypochlorite [1], and these combinations have been used to select *Mycobacterium* from microbial background. However, in some cases, they can also reduce the recovery of mycobacteria [73,74].

*Mycobacterium* spp. can be cultivated at room temperature or at environmental temperature, depending on the species, and take anywhere between 2 to 28 days to form clear colonies [75,76]. For example, *M. marinum* will grow at 30 °C, while others species such as *M. shottsii* and *M. pseudoshottsii* will grow at 23 °C and not well or at all at 30 °C [77]. *M. salmonifilum* is able to grow at 20–30 °C on specific media, and smooth colonies can be observed after 4–6 days [78]. *M. haemophilum* isolated on Middlebrook 7H10 agar cultivated at 29 °C [37]. Because *Mycobacterium* spp. are slow-growing organisms, it might be necessary to keep the culture plate about 2 to 3 months before discounting the possibility of a mycobacterial infection.

NTM can be identified with several biochemical reaction methods, including niacin accumulation test, arylsufatase test, nitrate reduction test, catalase test, citrate utilization tellurite reduction and growth in the presence of 5% NaCl (Table 2) [79].

### 6.2. Serological Diagnostics

*Mycobacterium* spp. can also be detected using immunohistochemistry on histological sections and immunohistochemical sections (IHC). According to Sarli et al. [80], immunostaining should be considered more sensitive than Ziehl–Neelsen (ZN), in particular in small and early granulomas, allowing the detection of *Mycobacterium* spp. 1 or 2 weeks post infection. However, non-specific inflammatory infiltration may be observed, especially in more active lesions, which can obscure the histological diagnosis of *M. marinum* infection [81].

In addition, NTM have been identified using Dot assays [82]. Moreover, several serological tests have been designed to diagnose and identify mycobacterial agents in humans and other mammals, such as the tuberculin skin tests or the Vollmer path test. These tests can cross-react with bacteria of the NTM complex, and could presumably be repurposed for their diagnostic [64,83].

### 6.3. Molecular Diagnostics

Several molecular diagnostic techniques have been developed to identify the *Mycobacterium* spp. associated with public health risk. The most common target for the identification of *Mycobacterium* is the 16S small subunit rRNA gene [3] and Taq-Man assay and SybrGreen. Moreover, RT-qPCR procedures are available to detect *M. marinum* based on this sequence [84]. It is noteworthy that these sequences are quite similar throughout the *Mycobacterium* genus and, therefore, these PCRs do not generally allow one to identify the bacterium at the species level [85]. Other genes have been suggested for the development of PCR primers, including the heat shock protein 65kD gene (*hsp 65*) [86], as summarized in Table 3.

LAMP (loop-mediated isothermal amplification) assays have also been designed for the identification of *Mycobacterium* spp. and *M. gordonae* in guppies (*Poecilia reticulate*) [87]. Similarly, this technique has been used for the identification of *M. marinum* complex based on the detection of *mrs*A gene, and this approach was shown to have a very high sensitivity (threshold detection of seven copies) [88]. HRMA (high-resolution melting analysis) has also been applied to rapidly diagnose *Mycobacterium* spp. infections in fish [89]. Salati et al. utilized the FRET (fluorescence resonance energy transfer) method to distinguish samples containing *M. marinum* strains from other *Mycobacterium* spp., and this method has been shown to allow the detection of *M. marinum* in fish farms even before the fish develop clinical signs [90]. More recently, the identification of mycobacteria (*M. fortuitum, M. chelonae,* and *M. abscessus*) has been performed using matrix-assisted laser desorption/ionization time-of-flight mass spectrometry (MALDI-TOF MS). This method is based on unique spectral fingerprints of extracted proteins and has the advantage of being comparatively fast and allowing identification at the species level. In addition, this method also provides data for phylogenic analysis [16,91,92].

## 7. Virulence Factors

Mycobacteria are well established as facultative intracellular pathogens, and mycobacteria infecting phagosomes can withstand the normal processes of acidification and phagolysosomal fusion [3,93]. Further research has moreover shown that, like is the case in *M. tuberculosis*, functional mammalian cell entry 4 (mce4) and mce1 gene clusters are required for *M. marinum* to gain entry into the host cells [1]. In addition, the bacterium is able to escape from phagosomes through the activity of the secretory protein ESAT-6 [94], both of which appear reminiscent of *M. tuberculosis*.

Moreover, signature-tagged mutagenesis (STM) has identified 33 putative virulence genes associated with the persistence of *M. marinum* in a goldfish model [95]. Notably, only 5 of these genes have homologues in *M. tuberculosis*, including pks (polyketide synthase) genes, genes belonging to the proline-proline-glutamic acid (PPE), family gene and a transcriptional regulator with an AraC signature [1,95].

Moreover, screening of a transposon-mutation library has shown that the cell wall-associated lipid PDIM, PGLs and the ESAT-6 secretion system 1 (ESX-1) are required for the survival of *M. marinum* in a zebrafish model [96,97]. PDIM and ESAT-6 have been previously described as important factors for infections in *M. tuberculosis* [98,99].

The ESAT-6 secretion system, labelled from 1 (ESX-1) to 5, is a type VII secretion system, that has been identified in *Mycobacteriaceae*. Among these, ESX-1 is considered an important virulence factor of *Mycobacteriaceae*, and has been associated with the secretion of multiple proteins, including the virulence factors EsxA (ESAT-6) and EsxB (CFP-10). ESAT-6 has been shown to play a role in the infection of *M. tuberculosis* [94], as well as in the escape from phagosomes. ESX-1 has also been shown to play a role in inducing the differentiation of macrophages into foam cells [100], which is necessary for the acquisition of LDLR by the bacterium. While not all members of the genus *Mycobacterium* possess a ESX-1 secretion system, it has recently been reported that ESX-4 could play a similar role in the regulation of intracellular growth and phagosomal escape [101].

The ESX-3 system has multiple functions; notably, it is central to the acquisition of iron and zinc. Slow-growing mycobacteria also express their own version of the ESX-5 secretion system that is only present in this group. More than 100 proteins of the Pro-Glu and Pro-Pro-Glu (PE and PPE) family have been shown to be transported through the ESX-5 system in *M. marinum*. Weerdenburg et al. reported that ESX-5 deficient *M. marinum* displayed significantly reduced virulence in zebrafish embryos, suggesting that ESX-5 gene is an important virulence factor [102].

In fast-growing mycobacteria, identified secretion systems consist of MspA-like porins for the uptake and transport of nutrients, such as glucose and serine, as well as the export of hydrophilic β-lactam antibiotics [103,104].

Another important secretion system of the *Mycobacteriaceae* (*M. tuberculosis*) is the accessory Sec translocation pathway [105] SecA2. Several proteins have been shown to be secreted through this pathway [106], including multiple proteins known to block the inhibition of the phagosome and autophagosome [107], and play a role in the survival of *M. tuberculosis* in macrophages. In *M. marinum*, inhibition of SecA2 resulted in a reduced export of several proteins, including the virulence factor protein kinase G a protein, known to interfere with the phagosome–lysosome fusion [108] to the cell membrane, as well as a hindered ability to form granulomas in a mouse model, as well as in zebrafish [109,110].

Mutations in the lipooligosaccharide (LOS) of *M. marinum* have shown that the significant truncation of this protein increased the elimination of this bacterium by macrophages in a Toll-like receptor 2 dependent manner, which suggested that these LOS play a role in the escape of NTM from the fish’s immune system [111]. More recently, Wu et al. have described the role of the transcription factor WhiB, which plays a role in the resistance of *Mycobacterium marinum* to oxidative stress and is required for intracellular replication in macrophages and virulence in a zebrafish model [111].

## 8. Treatment and Control of Mycobacteriosis in Fish

### 8.1. Vaccination against Mycobacteriosis in Fish

Vaccination against fish mycobacteriosis would be invaluable for the prevention and control of this disease, and several attempts have been performed over the years. Interestingly, the BCG vaccine (Bacillus Calmette and Guerin) was found to stimulate the expression of several immune genes in the Japanese flounder (*P. olivaceus*), including IL-1β, IL-6, IFN-γ TNF-α, and this vaccine was associated with increased survival upon challenge, although, unexpectedly, these authors did not report on antibody [112].

Injection with heat-killed *M. marinum* results in the secretion of IgM and TNF-α in European Seabass (*Dicentrarchus labra*), in association with decreased mortality against mycobacteriosis in fish [113]. Similarly, the injection of heat killed *M. bovis* was reported to provide cross-protection in zebrafish [114,115]. Moreover, the injection of rainbow trout with mycobacterial extracellular products (ECP) from various aquatic *Mycobacterium* spp. (strains TB40, TB267 or *M. marinum*) results in enhanced levels of phagocytes, lysozyme and antibodies, as measured by enzyme-linked immunosorbent assay and Western blot [116]. Moreover, the survival of fish injected intraperitoneally with high dose of *M. marinum* was also improved by immunization with the mycobaterial enzyme RpfE [117].

DNA vaccines have been designed targeting the secreted fibronectin-binding protein of *Mycobacterium* spp. Ag85A, and have resulted in the protection of hybrid-striped bass (*Morone saxatilis* × *Morone chrysops*) against *Mycobacterium* spp. including *M. marinum* [118,119]. Similarly, Pasnik et al. demonstrated that DNA vaccine can lead to the development of an immune response against *M. marinum* and reduce mortality of vaccinated fish (hybrid-striped bass) [120]. Moreover, the application of live attenuated *M. marinum* mutant (L1D) in zebrafish resulted in increased survival (more than 70% survival rate after 50 days) following challenge by the injection of a solution of *M. marinum* [121]. However, despite these promising developments, no vaccines are currently commercially available against mycobacteriosis in fish [122,123].

Finally, a more recent development is the increasing adoption of autogenous vaccines, tailor-made vaccines based on local isolate originating from the very site they are aimed to protect, these vaccines have several advantages, in particular that they can be made available for diseases that do not justify the cost of developing a commercial vaccine [124]. While we are not aware of such autogenous vaccines being used against NTM, the use of autogenous vaccines in Nile tilapia (*Oreochromis niloticus*) has recently been found to be protective against subsequent intraperitoneal injection with *Francisella noatunensis* subsp. *orientalis* (relative percent survival = 100% and significantly increased antibody titers) [125]. *Francisella noatunensis* represents some similarities with NTM, notably they are slow-growing facultative intracellular bacteria associated with granulomatous lesions, albeit they belong to the Gram negative. Therefore, this development can be encouraging regarding the application of autogenous vaccines against NTM in fish.

### 8.2. Antibiotherapy

Infections by *Mycobacteriaceae* have traditionally been treated through antibiotherapy and the antibiotics rifampicin, streptomycin, erythromycin, ethambutol, isoniazid, doxycycline, kanamycin, ethionamide, minocycline and tetracycline have encountered some success [2,126]. However, members of this bacterial genus are well known to absorb drugs only slowly, and to require prolonged treatment. The susceptibility of *Mycobacterium* spp. to antibiotics also varies between isolates and between fast and slow growing species: fast growing mycobacteria are more sensitive to tigecycline, tobramycin, clarithromycin and amikacin, while slow growing mycobacteria are susceptible to amikacin, clarithromycin, and rifampin [26].

Moreover, as is the case in most genus of pathogenic bacteria, acquired antibiotic resistance is an issue. For example, *M. fortuitum* isolated from aquaria in South Africa and zebrafish facility displayed resistance to macrolide and other antibiotics, such as streptomycin, isoniazid, rifampicin, and ethambutol. Moreover, different *M. marinum* strains (AR103K, OR932, TG19 and ATCC927) have been isolated from zebrafish, that were resistant to trimethoprim and sulfamethoxazole [26,127]. Conversely, one study suggested that kanamycin sulphate remains an effective antibiotic against *Mycobacterium* spp. in guppies (*Lebistes reticulatus*) [42,128]. Similarly, tigecycline and clarithromycin are efficacious to treat *M. chelonae* in zebrafish, and while these drugs may not totally eliminate the pathogen, they can reduce the severity of the disease [129].

In this situation, the best option for the control of mycobacteriosis remains prophylaxis. Because the bacterium is widespread in the environment, episodes of stress and immune suppression in the fish are a major factor in outbreaks of the disease. Therefore, farmers should be minimizing stress and poor water quality, alongside providing appropriate nutrition, and avoiding unnecessary handling. The quarantine of newly arrived fish can reduce the risk of disease outbreaks, especially for fish with clinical signs. However, many infected fish may not display obvious clinical signs of the disease, making this screening more difficult. The systemic sampling and testing of new fish using sensitive diagnostic methods such as PCR are therefore advisable whenever possible.

## 9. Conclusions

Mycobacteriosis is an important disease in fish, and is associated with infections that can cause high-levels of mortality, ranging from 10% to 100% of the infected fish. The most common path of transmission is considered to be the ingestion of contaminated material. Fish can also be infected via open wounds in their skin. Vertical transmission has also been described and can occur in fish through egg or sperm products.

It is likely that almost all fish species are susceptible to *Mycobacterium* spp., and several mycobacterial species have been associated with diseases in fish, in particular *M. marinum*, *M. chelonae* and *M. fortuitum*. These species are also infective for humans and have a potential for zoonotic infection. Mycobacteriosis in humans is often occupation-acquired, and particularly common in workers in fish aquaculture or fish industries, fishery professionals, and ornamental fish hobbyists; consumers are also susceptible. Clinical signs can occur in humans such as superficial skin lesions with crusted and deep tissue infections can be observed, including infection in tendons and bone.

Infected fish may display skin lesions or internal signs, such as enlarged spleen, liver, and kidney. Gray and white nodules can also often be observed in the internal organs. Infections limited to the skin and soft tissue must be distinguished from infections extending to deeper tissues. Diagnosis of *Mycobacterium* spp. can be difficult, and several identification procedures have been described, including several molecular and serological diagnostics have been developed for the identification of *Mycobacterium* spp., including PCR and immunoblotting. Notably, PCR methods do not allow for the identification of NTM at the species level. However, this has limited consequences from a practical standpoint, as the condition these bacteria cause and the treatments against them are similar between species. Consequently, knowing that infection with NTM is taking place is generally sufficient, and specific identification would not change the course of treatment. Even under these circumstances, the development of species-specific PCR primers would be advisable. Until then, the application of mass-spectrometry is a more discriminating tool, although it has the limitations of requiring equipment that is less commonly owned by clinical practices.

The pathogenesis and virulence of NTM remain poorly understood and still need more investigation although, *M. tuberculosis* and *M. marinum* share several virulence and pathogenicity mechanisms. For example, *M. marinum* can escape into the cytoplasm of infected macrophages and spread the infection from cell to cell. Another important feature of the bacterium’s virulence arsenal appears to be the various type VII secretion systems, in particular the ESX-1. Nevertheless, it is clear that much is still to be learned regarding the mechanisms of disease in fish, and more research is needed on that subject.

Because *Mycobacterium* spp. does not react very much to most antibiotics, the treatment of mycobacteriosis is difficult and prolong antibiotic therapy is generally required as well as depopulation of infected fish. Moreover, because the therapeutic arsenal against mycobacteriosis is limited, especially considering the risks associated with antibiotic resistance, the development of alternatives would be highly beneficial. In recent decades, probiotics and other feed supplements have become increasingly adopted by fish farmers [130,131]. They are often efficacious, although most of this research has been conducted on other pathogens than *Mycobacterium* spp. [132]. Similarly, while no vaccine are currently commercially available, it is likely that autogenous vaccines could be applied to protect fish stocks although, once again, this will require more investigation to confirm.

The advantage of the present review is to provide an up to date overview of NTM in aquatic animals and to try to keep a practical approach that is helpful for clinical practitioners. Its main limitation is the limited understanding regarding some aspects of this disease. NTM have only been the subject of limited research in comparison to how common they are; likely, this is an effect of them being slow-growers and difficult to cultivate.

## Figures and Tables

**Table 1:** *Mycobacterium* spp., aquatic host and zoonotic potential.

Species	Aquatic Host	Environment	Zoonotic Potential
*M. szulgai*	Crocodile	Fresh water	Yes
	African clawed frogs (*Xenopus tropica*)		
*M. septicum/*	Zebrafish (*Danio rerio*), Cichlid (*Pseudotopheus lombardoi)*, Koi fish	Fresh water	Yes
*M. peregrinum*	*Labidochromis caeruleus*, Black mollies (*Poecilia sphenops*), guppies (*Poecilia reticulata*), green swordtails (*Xiphophorus hellerii*), catfish (*Pangasius* *hypophthalmus*)		
*M. chelonae*	Multiple	Fresh and Marine water	Yes
*M. avium*	Dwarf Cichlid (*Apistogramma cacatuodes)*	Fresh water	Yes
*M. abscessus*	Zebrafish (*Danio rerio*)	Fresh water	Yes
	Medaka (*Oryzias latipes*)		
	Milkfish (*Chanos chanos*)		
	German blue ram (*Mikrogeophagus ramirezi*)		
*M. haemophilum*	Zebrafish (*Danio rerio*)	Fresh water	Yes
*M. lentiflavum*	Swordtail *(Xiphophorus hellerii)*	Fresh water	Yes
*M. gordonae*	Goldfish (*Carassius auratus*)	Marine water	Yes
	Guppy (*Poecilia reticulate)*		
	Angel fish (*Pterophyllum scalare*)		
*M. chesapeaki*	Striped bass (*Morone saxatilis)*	Marine water	Unknown
*M. marinum*	Multiple	Fresh and marine water	Yes
*M. montefiorence*	Moray eel (*Gymnothorax funebris)*	Marine water	Unknown
*M. neoaurum*	Chinook salmon (*Oncorhynchus tschawytscha*)	Marine water	Yes
*M. shottsii*	Striped bass (*Morone saxatilis)*	Freshwater	Unknown
*M. pseudoshottsii*	Yellow tail (*Seriola quinqueradiata*), Greater amberjack (*Seriola dumerili*), Striped jack (*Pseudocaranx dentex*)	Marine water	Unknown
*M. fortuitum*	*Neon tetra (Paracheirodon innesi)*	Freshwater	Yes
*M. syngnathidarum*	syngnathid fish	Marine water	Unknown
*M. flavescens*	Tiger Oscar *Astronatus ocellatus*	Fresh water	Yes
*M. smegmatis*	*Poecilia sphenop, Poecilia reticulata, Gymnocorymbus ternetzi*	Fresh water	Yes
*M. nonchromogenicum*	*Betta splendens, Carassius auratus, Poecilia reticulata, Pterophyllum scalare*	Fresh water	No
*M. stephanolepidis*	Filefish (*Stephanolepis cirrhifer*)	Fresh water	Unknown
*M. holsaticum*	Silver moony fish *(Monodactylus argenteus)*	Marine water	Yes
*M. salmoniphilum*	Burbot (*Lota lota)*	Fresh water	No

**Table 2:** Biochemical test for the identification of *Mycobacterium* sp.

Biochemical Tests	Results
Growth in NaCl 5%	Negative (*M. gordonae, M. ulcerans, M. kansasii, M. marinum, M. simiae, M. szulgai, M. scrofulaceum, M. xenopi, M. avium, M. intracellulare, M. chelonae, M. diernhoferi, M. celatum, M. terrae)*
Arylsulfatase	Negative (*M. avium, M. intracellulare, M. smegmatis, M. kansasii, M. simiae, M. szulgai, M. scrofulaceum, M. asiaticum, M terrae, M. gordonae*) (weakly positive after 3 day)
Catalase	Positive (*M. fortuitum, M. chelonae, M. abscessus, M. smegmatis, M. kansasii, M. marinum, M. ulcerans, M. simiae, M. szulgai, M. scrofulaceum, M. gordonae)*
Nitrate reduction	Negative (*M. avium, M. intracellulare, M. chelonae, M. abscessus, M. ulcerans, M. simiae, M. scrofulaceum, M. gordonae, M. xenopi, M. celatum)*
Urease activity	positive (*M. marinum*, *M. fortuitum*, *M. chelonae*, *M. abscessus, M. kansasii, M. simiae, M. szulgai, M. scrofulaceum, M. flavescens)*
Pirazinamidase	Positive
Thiophene-2-carboxylic hydrazide	Positive

**Table 3:** PCR primers used for the identification *Mycobacterium spp*.

Target Gene	Primer Name	Sequence (5′ to 3′)
*dnaJ1*	J10F	CGIGARTGGGTYGARAARG
	J335R	ARICCICCGAAIARRTCICC
*secA1*	MtuF1	GACAGYGAGTGGATGGGYCGSGTGCACCG
	MtuR3	ACCACGCCCAGCTTGTAGATCTCGTGCAGCTC
32-kDa protein genes	MV1	GGCCAGTCAAGCTTCTACTCCGACTGG
	MV2	GCCGTTGCCGCAGTACACCCAGACGCG
*hsp65* (heat-shock protein 65)	21M13F	ACCAACGATGGTGTG TCCAT
	21M13R	CTTGTCGAACCGCATACCCT
	Tb11	ACCAACGATGGTGTGTCCAT
	Tb12	CTTGTCGAACCGCATACCCT
*erp* (exported repeated protein)	erp-C1	GCTCTA GACGAGCGGTCATCGGTTGCATAGGG
	erp-C2	GCTCTAGATTAGGCGACCGGCACGGTGATTGG
	erp-C3	CGGAATTCATGGTGCTCGGGCCGCTC
	erp-C4	CGGAATTCACCCAGG CCGCGCTGGTCACC
	erp-C6	GCTCTAGATCAGGCAGGCGGCGGCACGGGTGC
	erp-C5	CGGAATTCAAAC AAGCAGCATCGATAGCC
	erp-C7	GCTCTAGACTAC GTGACAGGAATCAGTGATAT
	erp-8	GTGCCGAACCGACGCCGACG
	erp-9	GGCACCGGCGGCAGGTTGATCCCG
*rpo*B (RNA polymerase B subunit)	MycoF	GGCAAGGTCACCCCGAAGGG
	MycoR	AGCGGCTGCTGGGTGATC ATC
	RPO5′	TCAAGGAGAAGCGATACGA
	RPO3′	GGATGTTGATCAGGGTCTGC
rec A	recF1	GGTGGTCGNCTANTGTGGTG
	recR1	AGCTGGTTGATGAAGATYGC
	recF2	GYGTCACSGCCAACCGAY
	recR2	TTGATCTTCTTCTCGATCTC
	recF3	GGCAARGGYTCGGTSATG
*sodA*	sodlgF	GAAGGAATCTCGTGGCTGAATAC
	sodlgR	AGTCGGCCTTGACGTTCTTGTAC
*erm* (Erythromycin ribosome methyltransferase gene)	ermF	GACCGGGGCCTTCTTCGTGAT
	ermR1	GACTTCCCCGCACCGATTCC
16S-23S internal transcribed spacer (ITS)	ITS-F	CCTTTCTAAGGAGCACC
	ITS-R	GATGCTCGCAACCACTATCC
16S rRNA	T_39_	GCGAACGGTGAGTAACACG
	T_13_	TGCACACAGGCCACAAGGGA
	T_43_	AATGGGCGCAAGCCTGATG
	T_531_	ACCGCTACACCAGGAAT

## Abbreviations

16S rRNA: 16S ribosomal RNA; NTM: Non-tuberculous mycobacteria; ESAT-6: 6 kDa early secretory antigenic target; CFP-10: 10 kDa culture filtrate antigen; IHC: Immunohistochemical; ZN: Ziehl-Neelsen; RT-qPCR: real-time quantitative polymerase chain reaction; LAMP: loop-mediated isothermal amplification; HRMA: high-resolution melting analysis; FRET: fluorescence resonance energy transfer; MALDI-TOF MS: matrix-assisted laser desorption/ionization time-of-flight mass spectrometry; ECP: extracellular product; TNF-α: tumor necrosis factor alpha; RpfE: Resuscitation Promoting factors; mce: mammalian cell entry; STM: signature-tagged mutagenesis; PPE: proline-proline-glutamate; pks: Polyketide synthase; IL-6: interleukin-6; PDIM: phthiocerol dimycocerosates; PGLs: phenolic glycolipids; LOS: lipooligosaccharides; *hsp65:* heat-shock protein 65; *erp*: exported repeated protein; *rpo*B: RNA polymerase B subunit; *erm*: Erythromycin ribosome methyltransferase gene.
